# An antireflection transparent conductor with ultralow optical loss (<2 %) and electrical resistance (<6 Ω sq^−1^)

**DOI:** 10.1038/ncomms13771

**Published:** 2016-12-19

**Authors:** Rinu Abraham Maniyara, Vahagn K. Mkhitaryan, Tong Lai Chen, Dhriti Sundar Ghosh, Valerio Pruneri

**Affiliations:** 1 ICFO–Institut de Ciències Fotòniques, The Barcelona Institute of Science and Technology, Av. Carl Friedrich Gauss, 3, Castelldefels, 08860 Barcelona, Spain; 2 ICREA—Institució Catalana de Recerca i Estudis Avançats, Passeig Lluís Companys, 23, 08010 Barcelona, Spain

## Abstract

Transparent conductors are essential in many optoelectronic devices, such as displays, smart windows, light-emitting diodes and solar cells. Here we demonstrate a transparent conductor with optical loss of ∼1.6%, that is, even lower than that of single-layer graphene (2.3%), and transmission higher than 98% over the visible wavelength range. This was possible by an optimized antireflection design consisting in applying Al-doped ZnO and TiO_2_ layers with precise thicknesses to a highly conductive Ag ultrathin film. The proposed multilayer structure also possesses a low electrical resistance (5.75 Ω sq^−1^), a figure of merit four times larger than that of indium tin oxide, the most widely used transparent conductor today, and, contrary to it, is mechanically flexible and room temperature deposited. To assess the application potentials, transparent shielding of radiofrequency and microwave interference signals with ∼30 dB attenuation up to 18 GHz was achieved.

## Abstract (web summary)

Transparent conductors are fundamental for optoelectronics. Using the transfer matrix method to optimise a multistructure of anti-reflection coatings containing an ultrathin metal film, Maniyara *et al*. achieve the highest transmittance of an antireflection transparent conductor combined with low resistance.

A wide range of electronic and optoelectronic devices require transparent conductors (TCs) to function. These include, for example, displays, light-emitting diodes and photovoltaic cells, where TCs are used to either apply or collect electrical signals without reducing optical transmission (*T*)^1^,^2^,^3^,^4^,^5^. An intensive effort has been devoted to search for TC materials that can replace indium tin oxide (ITO), a wide band gap semiconductor, which is used in most of, if not all, the devices today. Despite possessing large *T*, low electrical sheet resistance (*R*_s_), high chemical and environmental stability, ITO requires high temperature processing, has poor mechanical flexibility and high raw material cost^6^. Among the material alternatives, Al-doped ZnO (AZO), carbon nanotubes, metal nanowires, ultrathin metals, conducting polymers and, most recently, graphene have been extensively considered^7^,^8^,^9^,^10^,^11^,^12^,^13^,^14^,^15^,^16^,^17^,^18^,^19^,^20^. Some of these alternatives can overcome the mechanical fragility, high temperature processing and/or cost of ITO, but still suffer from one or more drawbacks such as poor adhesion, large surface roughness and high optical scattering, and not always achieve a competitive trade-off between *T* and *R*_s_^21^,^22^,^23^,^24^,^25^.

Ultrathin metal films (UTMFs) can present very low *R*_s_ but their *T* is low unless antireflection (AR) undercoat and overcoat layers are applied^26^,^27^. Although the AR concept in TC is widely known, it has never been investigated and exploited fully, especially in conjunction of high-quality UTMFs. In the absence of scattering, which is the case of the work presented in this study, the optical loss (OL) that accounts for the reduction in *T* with respect to the bare substrate (without TC) comes from absorption (*A*) of the TC material itself and reflection (*R*) at the interfaces in the TC on substrate structure. Here we study in-depth the AR properties of a TC structure on glass made of ultrathin Ag, TiO_2_ undercoat and AZO overcoat layers, and show that, through a proper optimised design, reflection can be strongly suppressed. The OL of the optimized TiO_2_/Ag/AZO structure (∼1.6%) is even lower than that of a single layer graphene (2.3%), whereas the figure of merit (FoM) is four times larger than that of ITO, thanks to the very high *T* (>98%) and low *R*_s_ (<6 Ω sq^−1^). The proposed TC has the highest electro-optical performance (FoM) reported so far, is mechanically flexible, room temperature processed and its potential for real applications is demonstrated by showing that it can be used as an efficient transparent shield for radiofrequency and microwave electromagnetic interference (EMI) signals, with 30 dB attenuation up to 18 GHz.

## Results

### Structure and optical performance of AR-TC electrode

**Figure 1:**
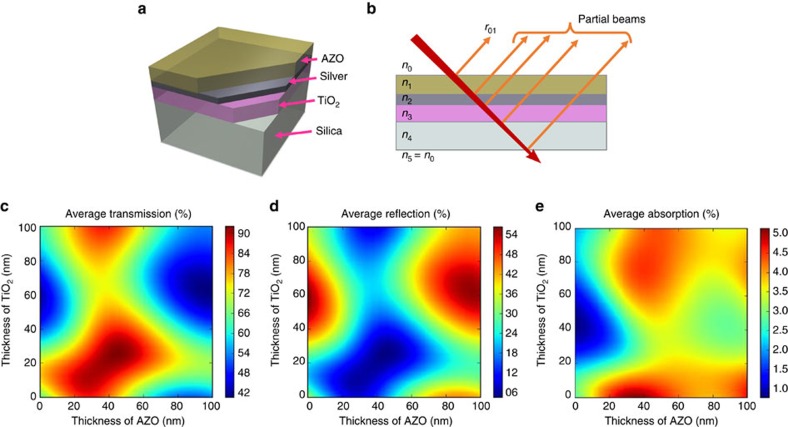
Structure and modelling of AR-TC electrode. (**a**) Structure of AR-TC. (**b**) Conceptual diagram showing multiple reflection contributions leading to destructive interference and AR effect. Simulated (**c**) transmission, (**d**) reflection and (**e**) absorption of AR-TC for different TiO_2_ and AZO thicknesses. For all the structures, the Ag film thickness is kept constant at 12 nm. The transmission, reflection and absorption include the substrate contribution, that is, they refer to the whole TC on substrate structure, and are average values over 400–700 nm wavelength range.

The structure of the proposed multilayer AR-TC is shown in Fig. 1a. For the experiments and simulations, we deposited and studied in detail the AR-TC structure on a fused silica substrate, but the work can be extended to other transparent substrates with similar refractive index, including other glasses and polymers. In particular we will also show some preliminary results on Corning Eagle XG glass and poly ethylene terephthalate (PET). Among the metals, we chose Ag as it has among the highest electrical conductivity and lowest absorption loss. However, it has a high reflection and tends to grow in an island form at small thicknesses. Previous works showed that proper seed layers favour nucleation of Ag films, which became continuous for thicknesses much lower than those when they were directly deposited on the substrate's surface^28^. Oxide undercoat and overcoat layers can reduce the reflection of Ag^29^.

TiO_2_ is an ideal undercoat material as it has a high refractive index (high AR effect), promotes strong film adhesion to the substrate, chemical stability and nucleation seeding properties. AZO has been used as an overcoat layer because of its relatively low refractive index and the fact that its low conductivity ensures electrical contact between the Ag film and other materials, which is essential for the functionality of several devices incorporating the AR-TC.

The AR effect in a multilayer structure relies on destructive interference between light reflected at the different interfaces. This can be understood using the generalized Fresnel equation for the reflection of the multilayer structure, which is given by^30^:


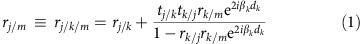


where *r*_*j*/*m*_ is the reflection coefficient for a stack starting at layer *j* and ending at layer *m*, and *k* identifies any intermediate layer; 
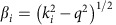
 and *q*=*ω* sin(*θ*)/*c* are the perpendicular and the parallel components of the wave vector in the layer *i*, respectively, with dielectric permittivity *ɛ*_*i*_, magnetic permeability *μ*_*i*_ and thickness *d*_*i*_. *k*_*i*_=*n*_*i*_*ω*/*c*, where *n*_*i*_=(*ɛ*_*i*_*μ*_*i*_)^1/2^ is the refractive index of the layer, and *ω* and *c* are the frequency and the speed of the light in vacuum, respectively. For the structure of our work shown in Fig. 1b, one can write:


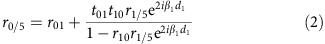


which accounts for total reflection at the top surface (layer number 1 is considered the intermediate layer). Here, *r*_1/5_ is the total reflection coefficient of the layers from one to five and the interface reflection and transmission coefficients between any adjacent layers are:


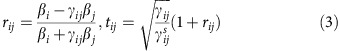


with 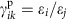 and 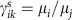 for *p*- and *s*-polarized light, respectively.

The reflection suppression is given by:


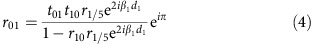


It is noteworthy that the π phase term is related to the destructive interference condition. In this formula, *r*_01_ represents the primary reflected beam at the top interface, whereas the multiple reflected beams are merged into a single term on the right-hand side of the equation. One can easily verify this by reapplying the generalized Fresnel formula of the multiplayer *r*_1/5_ and use the geometric series representation of the denominator


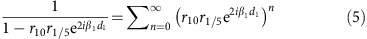


In this way, if need be, all the multiple reflection terms can be identified.

Maximum AR corresponds to minimum *r*, which can be obtained through optimization of film's thicknesses, as in our case materials are predefined. To determine the optimum thickness of each layer, transfer matrix method (TMM) was used. TMM automatically takes into account multiple reflections of a multi-layer structure and determines the optical response of the system including the entire structure's transmission and reflection, together with absorption in each layer. In our experiments, the thickness of Ag was kept constant at a thickness (12 nm) that provides low *R*_s_ (<6 Ω sq^−1^), whereas TiO_2_ and AZO thicknesses were varied to find the optimal combination for minimum *R*. Figure 1c–e show the optical simulation results with clear dependence of *T*, *R* and *A* on TiO_2_ and AZO thicknesses. It is noteworthy that the simulated parameters include the substrate's contribution, that is, they refer to the entire multilayer TC on substrate structure. This means that in the case of complete AR, the simulated *R* would not tend to zero but the value corresponding to the backside substrate–air interface (∼3.5%). Simulation results point out that maximum *T* and minimum *R* should be obtained for TiO_2_ and AZO thicknesses in the range of 20–30 nm and 30–50 nm, respectively. Under these conditions, the absorption takes almost entirely place in the silver layer (Supplementary Fig. 1). Similar trends can be observed for Corning Eagle XG glass and PET polymer substrates, which have a refractive index different from fused silica (Supplementary Fig. 2).

**Figure 2:**
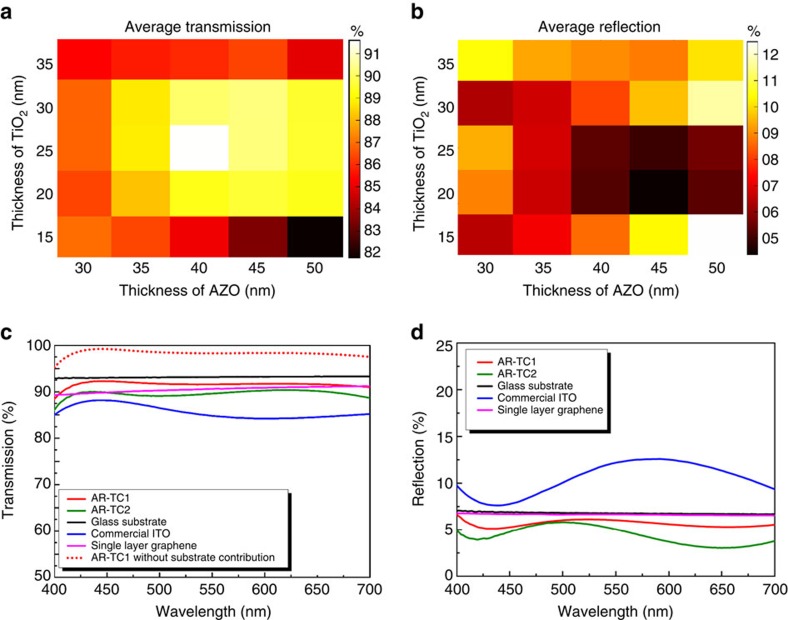
Optical performance of AR-TC electrode. Experimental average values of (**a**) transmission (*T*_AVE_) and (**b**) reflection (*R*_AVE_) over the visible wavelength range (400–700 nm) of the proposed AR-TC, for varying thickness of TiO_2_ and AZO (25 different samples were prepared and measured. Each square corresponds to a sample with the oxide thickness indicated). Wavelength dependent (**c**) transmission and (**d**) reflection of optimal AR-TC (AR-TC1 and AR-TC2) compared with bare fused SiO_2_ substrate, single-layer graphene and commercial ITO. Measured values include substrate contribution and two side reflections. The dashed line in **c** corresponds to the transmission of AR-TC1 without the substrate contribution, that is, the ratio between the AR-TC1 transmission and glass substrate transmission (continuous lines). The average TC transmission is calculated to be 98.33%.

**Figure 3:**
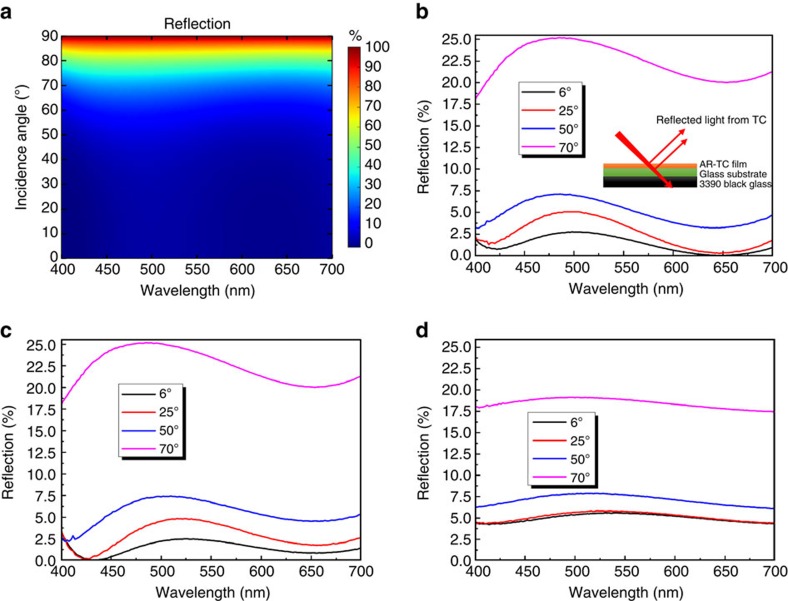
Single-side angular-dependent reflection performance of AR-TC electrode. (**a**) Simulated angle-dependent *R* of AR-TC 1 for varying angle of incidence. Experimental angle-dependent one surface *R* of (**b**) AR-TC 1, (**c**) AR-TC 2 and (**d**) commercial ITO for incidence angles of 6°, 25°, 50° and 70°. Inset of **b**: scheme of one-side reflection measurement obtained by index matching a completely absorbing material (black glass) to the back surface.

Figure 2a,b show the experimental results of *T*_AVE_ and *R*_AVE_, average values of *T* and *R*, respectively, over the visible wavelength range (400–700 nm), for varying thicknesses of TiO_2_ and AZO. There is a strong agreement with the simulation. The optimum structures were TiO_2_ (25 nm)/Ag (12 nm)/AZO (40 nm) and TiO_2_ (20 nm)/Ag (12 nm)/AZO (45 nm), named AR-TC1 and AR-TC2, respectively (we have indicated in parenthesis the layer's thicknesses). Figure 2c,d show the measured wavelength-dependent *T* and *R* spectra of AR-TC1 and AR-TC2, respectively, compared with bare fused SiO_2_ substrate, commercial ITO (∼135 nm thick) and single-layer graphene on fused silica. *T*_AVE_ for the investigated AR-TC structures was as high as 91.6%, ∼5% larger than that of ITO (86.7%) and very close (only ∼1.6% lower) to that of the bare fused silica substrate (93.2%). It is worth noting that Fig. 2d includes measurements of two side *R*. To verify the AR quality of the proposed AR-TC, we have also measured single side *R*. The inset in Fig. 3b illustrates the method of measuring it. The back *R* from the substrate–air interface has been suppressed by adding an absorbing substrate (black glass), index matched to the sample through an appropriate oil. The residual *R* from the AR-TC, with an average value of ∼1% in the 400–700 nm wavelength range, is low compared with both the uncoated substrate surface (3.5%) and ITO-coated surface (5%; Supplementary Fig. 3). This strong AR effect increases the value of *T* and reduces the OL for AR-TC compared with bare substrate to less about 1.6%, that is, even lower than that of a single-layer graphene. For a more complete assessment of the AR properties, we simulated (Fig. 3a) and measured (Fig. 3b) single side *R* for the AR-TC1 at different angles of incidence. Fig. 3c,d show the results for AR-TC2 and commercial ITO samples, respectively. The superior AR behaviour of the proposed AR-TC structure is maintained up to large angles (50°). It is noteworthy that preliminary experiments performed on Corning Eagle XG and PET substrates with same layers as AR-TC1 on fused silica indicate that even for these materials OL is already very low and transmission of TC very high (see Supplementary Table 1), despite the fact that the structures were not optimized to match a different refractive index.

### Electrical and mechanical properties

The *R*_s_ of all the AR-TC structures were around 5.75 Ω sq^−1^, less than half of the value of reference commercial ITO (14.01 Ω sq^−1^). It was almost entirely provided by the Ag layer. From an electrical point of view, the thicker the Ag layer the lower the *R*_s_. From an optical point of view, without the AR undercoat and overcoat layers, the thicker the Ag layer the larger the *R* and *A*, that is, the OL. However, the use of AR layers help to contain the increase of *R* as the layer gets thicker. There is thus an optimum trade-off for the Ag thickness, which was 12 nm in our case. The root mean square (RMS) surface roughness of the Ag film was measured to be 2.2 nm, much smaller than that when directly deposited on the fused silica substrate (6.5 nm). This confirms the importance of the undercoat TiO_2_ layer to achieve a very continuous and smooth Ag film, which ensures high electrical and optical performance of the TC structures.

For better comparing our AR-TC with ITO, graphene and other TCs in the literature, we used a widely accepted 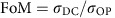) that is the ratio between direct current (DC) conductivity (*σ*_DC_) and optical conductivity (*σ*_OP_), which are related to *T* and *R*_s_^31^ as


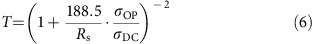


or


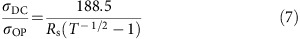


It is worth noting that in our estimates we considered *T* as the average value over the visible wavelength region (400–700 nm) of the entire TC on substrate structure. Our AR-TC exhibits *σ*_DC_/*σ*_OP_ of 730, 4 times greater than reference commercial ITO (180) and 95 times greater than single-layer graphene with relatively high doping (*R*_s_=325 Ω sq^−1^).

For a more straight comparison with other works in literature, we also used another common FoM, that is, Haacke^32^, given by


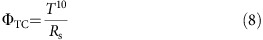


Table 1 shows the two FoMs (*σ*_DC_/*σ*_OP_ and Φ_TC_) of different TCs having high transmission reported in the literature^33^,^34^,^35^,^36^,^37^,^38^,^39^, which is re-calculated for transmission at 550 nm and includes substrate's contribution. A comparison with the literature of *T* (at 550 nm) as a function of *R*_s_ is in Table 1 and are shown in Supplementary Fig. 4. It is clear that, among all different TCs using different structures and materials, the proposed AR-TC has the highest FoM.

Mechanical flexibility is an important attribute of TC, for two main reasons. Flexible and foldable electronic/optoelectronic devices undergo strong curvature effects, whereas low-cost production requires roll-to-roll processing of large substrates coated with TC^40^. To demonstrate the flexibility of our AR-TC, we deposited it onto PET polymeric substrates and subjected it to continuous bending. Alongside, ITO-coated PET was also tested. *R*_s_ was measured, whereas the bending radius was varied from 10 to 3 mm. Supplementary Fig. 5 shows the mechanical flexibility results of proposed AR-TC compared with ITO. The AR-TC shows excellent flexibility due to the mechanical ductility of the Ag film, whereas ITO breaks due to its fragility. The corresponding change in *R*_s_ for the AR-TC structures is ∼12.7% after 1,000 cycles of bending with radius of curvature between 10 and 3 mm. Instead, the *R*_s_ of ITO increases 1,120% even for an order of magnitude smaller number of cycles (100).

### Performance in EMI shielding applications

To assess the application potential of developed AR-TCs, we have focused on transparent EMI shielding particularly important for display and imaging camera. An example is the possibility to realize invisible windows that attenuate micro-waves and transmit visible light. For such a window, not only the high visible optical transmission and very low electrical resistance (high shielding effect) are important, but equally crucial to achieve invisibility is the very low reflection that we have demonstrated. The EMI shielding efficiency (SE) quantifies the conductive coating's attenuation of electromagnetic radiation and is expressed by the ratio in decibels (dB) between incident power (*P*_i_) and transmitted power (*P*_t_):


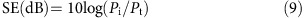


**Figure 4:**
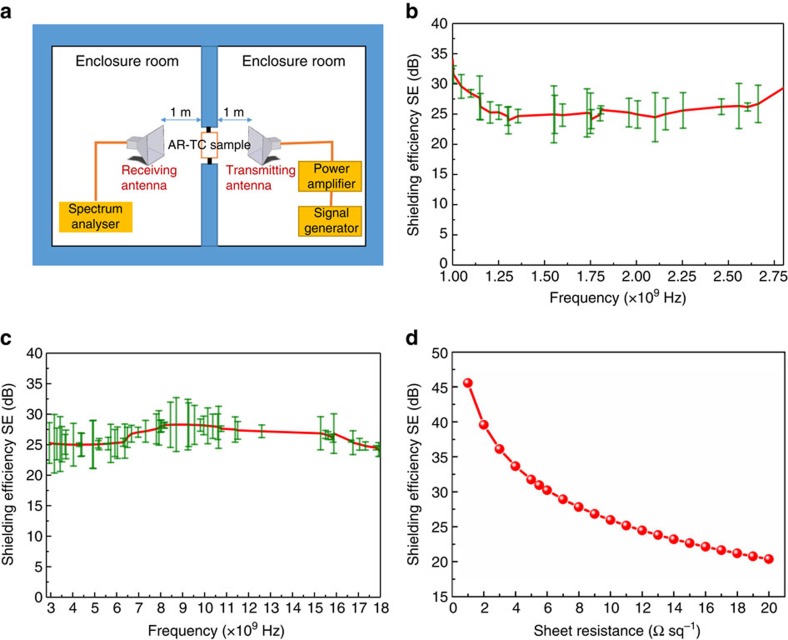
EMI shielding application of AR-TC electrode. (**a**) Scheme of EMI SE measurement setup with enclosure, transmitting and receiving antenna. (**b**) SE (attenuation) for AR-TC with *R*_s_ of ∼7 Ω sq^−1^ in 1–2.8 GHz and (**c**) 2.8–18 GHz. The error bars represent the difference between measured data and their average calculated using Fast Fourier Transform filtering. (**d**) SE as a function of sheet resistance (*R*_s_).

For example, an SE of 30 dB means that the conductive coating attenuates 99.9% of the incident power. The SE of the TC structures was measured in shielded room enclosure, so that errors caused by external signals were minimized. For the measurements, a 7 × 7 cm AR-TC sample with *R*_s_ of ∼7 Ω sq^−1^ was prepared and properly located in an aluminium frame (see Supplementary Fig. 6). Figure 4a shows the measurement setup. A reference measurement with Al frame (without AR-TC) was carried out to be able to evaluate the SE of the AR-TC-coated glass window only.

Most of the studies of EMI shielding in the literature have been carried out in the X-band (8.2–12.4 GHz) as defence tracking, telephone microwave relay systems, weather radar, satellite communication and TV picture transmission work in this spectral region^41^. However, extending the frequency range is often of interest, for example, for modern house hold appliances, microwave heating, mobile phones, wireless communication equipment and satellite navigation. The EMI shielding in our work was evaluated with Horn antenna from 1–18 GHz.

Figure 4b,c show the average SE of AR-TC from 1–2.8 GHz and 2.8–18 GHz, respectively. It is evident from the figure that the AR-TC provides an efficient shielding in higher frequencies. An average 26.2 dB SE was measured with peak values well exceeding 33 dB. Importantly, in the widely used X band, the proposed AR-TC shows a high average SE (27.7 dB). SE would be even higher for the lowest *R*_s_ samples demonstrated in this work. This is depicted in Fig. 4d, according to the relation:


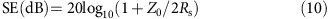


where *Z*_0_ is the free space impedance (377 Ω)^42^. For the AR-TC structure with *R*_s_ of 5.75 Ω sq^−1^, an SE of 30.8 dB is expected. To our knowledge, this is the highest SE reported for a TC with *T* including the substrate >90%. For comparison, state-of-the-art EMI shielding with ITO provides an SE of ∼25 dB with *T* <87%. The SE of other TCs, such as carbon nanotube, graphene and conductive polymers are even lower (<25 dB)^43^,^44^. Metal mesh can provide higher SE at the expense of transparency (visibility), as they typically have significant scattering (haze). They also present high reflectivity, contrary to the proposed AR-TC structures.

## Discussion

Our work exploits fully AR effect in optimized UTMF-based TCs. Through simulation and experiments, we have shown that destructive interference in a multilayer TC structure can lead to optical transmission >98% in the visible still keeping very high electrical conductivity (low electrical sheet resistance of 5.75 Ω sq^−1^). The resulting OL is even lower than single-layer graphene and the record FoM is four times larger than commercially available ITO. In addition, the proposed structure is haze free, highly adhered to the substrate, environmentally stable, mechanically flexible, room temperature deposited and its performance has been tested in EMI shielding with high attenuation.

## Methods

### AR-TC fabrication

Double-side, optically polished, ultraviolet-fused silica glass substrates, with a thickness of 1 mm and an area of 1 inch square were used as substrate. Before TC deposition, the substrates were cleaned in acetone followed by ethanol in ultrasonic bath, each process lasting 10 min. The substrates were then rinsed in deionized water and dried with nitrogen gas. The entire TC structure was deposited by magnetron sputtering without breaking the vacuum. The sputtering chamber was initially evacuated to a base pressure of ≈10^−7^–10^−8^ Torr. The target to substrate distance was maintained at 30 cm. The substrate holder was rotating during deposition with a speed of 60 r.p.m. For improving the adhesion properties of the film to the substrate, low power argon plasma cleaning was performed for 15 min inside the sputtering equipment before TC deposition. Bias power (40 W) and pressure (8 mT) were used for cleaning in Ar (20 sccm) atmosphere. TiO_2_ and Ag were deposited in pure Ar atmosphere, whereas AZO (3% Al doping) was deposited in an Ar/O_2_ mixture (flux ratio of 18:2), all of them at room temperature. An Ag (99.99%) target was used for depositing Ag films with DC power of 100 W and working pressure of 2 mTorr. The TiO_2_ film was deposited in radio frequency mode (150 W radio frequency power) at a pressure of 2 mTorr. The AZO film was deposited in the same condition but with a pressure of 1.4 mTorr. The deposition rate was 0.1, 3.5 and 0.3 Å s^−1^ for TiO_2_, Ag and AZO, respectively.

### Device characterization

The electrical properties of the films were measured using four-point method with cascade Microtech 44/7 S 2749 probe station connected to a Keithley 2001 multimeter. Typically, six measurements were performed at different positions and *R*_s_ was an average of the corresponding values. Agilent cary5000 UV-Vis-NIR Spectrophotometer with universal measurement accessory and polarizer was used for optical transmission measurements. Before measurements, the samples were cleaned using a TX 609 Technicloth wiper dampened with HPLC-grade reagent alcohol. A background scan was performed before each new measurement configuration (that is, polarization). Transmission and reflection (two surface) were taken without moving the sample, with the coated surface towards the incident beam, at 6°, 25°, 50° and 70°, for *s* and *p* polarizations. First, surface reflection was measured with an index matching oil on the back surface of the sample to a 3,390 black glass. In this way the second (back) surface reflection was completely suppressed. Flexibility tests were performed using a two-point bend testing setup connected to a motor driven by an electronic controller, allowing the arm to move back and forth along the horizontal direction. AR-TC deposited onto PET polymeric substrates and subjected it to continuous bending. *R*_s_ was measured while the bending radius was varied from 10 to 3 mm.

### EMI shielding performance demonstration

Attenuation of the AR-TC was measured using Horn Antennas that transmits and receive in the 1–18 GHz frequency range. The transmitting and a receiving Horn antennas were placed in two enclosure rooms, to minimize noises from external signals. Both antennas are separated 1 m away from the sample. Before measurements, the sample was prepared on 7 × 7 cm glass substrate, which was enclosed in 19.6 × 19.6 cm aluminium frame. The test was performed in two stages. In the first stage, reference attenuation measurement was carried out with only Al frame for establishing the Al frame contribution to SE. In the second stage, the same measurement was carried out with the AR-TC in the frame. Finally, effective attenuation was calculated by subtraction.

### Simulation methods

All simulations were performed using a TMM implemented in a python environment.

### Data availability

The data that support the findings of this study are available from the authors upon request.

## Additional information

**How to cite this article**: Maniyara, R. A. *et al*. An antireflection transparent conductor with ultralow optical loss (<2%) and electrical resistance (<6 Ω sq^−1^). *Nat. Commun.* **7**, 13771 doi: 10.1038/ncomms13771 (2016).

**Publisher's note:** Springer Nature remains neutral with regard to jurisdictional claims in published maps and institutional affiliations.

## Supplementary Material

Supplementary Information Supplementary Figures and Supplementary Table.

## Figures and Tables

**Table 1:** AR-TC performance comparison with other works.

Reference	Structure	Transmission (%) at 550 nm	Sheet resistance (Ω sq^−1^)	Haacke FoM ( × 10^−3^ Ω^−1^)	*σ*_DC_>*σ*_OP_ FoM
This work	TiO_2_/Ag/AZO	91.6	5.75	72.3	730.0
3	Cu_2_O/Cu/Cu_2_O mesh	88.1	15.1	18.6	189.0
29	TiO_2_/Ag/ITO	88.6	6.20	48.5	497.8
31	Dip-coated AgNw	89.9	10.2	34.0	339.0
33	ZnO/AgNw/AZO/ZnO	87.3	11.3	22.8	237.5
34	Graphene–metallic grid hybrid	90.0	20.0	17.4	173.9
35	Cu nanowire	90.0	25.0	13.9	139.1
36	Polymer–metal hybrid	89.4	10.0	32.6	327.0
37	ZTO/Ag/ZTO	83.2	8.8	18.0	222.2
38	Capillary printed AgNW	90.4	19.4	18.8	175.1
39	Doped single-layer graphene	86.4	325	0.71	7.69
	Commercial ITO	86.6	14.0	16.9	180.1

*σ*_DC_, direct current conductivity; *σ*_OP_, optical conductivity; Ag, silver; AgNw, silver nanowire; AR, antireflection; AZO, aluminium zinc oxide; Cu_2_O, copper oxide; Cu, copper; FoM, figure of merit; ITO, indium tin oxide; TC, transparent conductor; TiO_2_, titanium oxide; ZnO, zinc oxide; ZTO, zinc tin oxide.

FoMs are re-calculated for transmission at 550 nm and including substrate contribution, this being made of fused silica. FoM of different TCs compared with AR-TC. Both Haacke and DC to optical conductivity ratio FoMs are used.
