# Supplementary material for: An antireflection transparent conductor with ultralow optical loss (<2 %) and electrical resistance (<6 Ω sq−1)
Source: Nat Commun. 2016 Dec 19;7:13771. doi: 10.1038/ncomms13771 (PMC5187436; doi:10.1038/ncomms13771)
Supplement: Supplementary Information — Supplementary Figures and Supplementary Table. [file ncomms13771-s1.pdf]

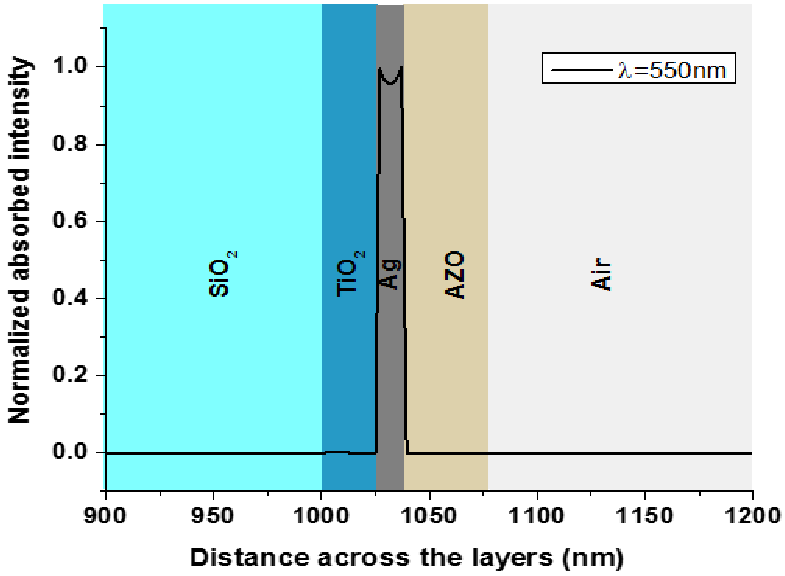

Supplementary Figure 1. Absorption Distribution. Simulated normalized absorption distribution in the multilayer AR-TC1 structure which clearly shows that most of the absorption, if not all, occurs in the Ag layer.

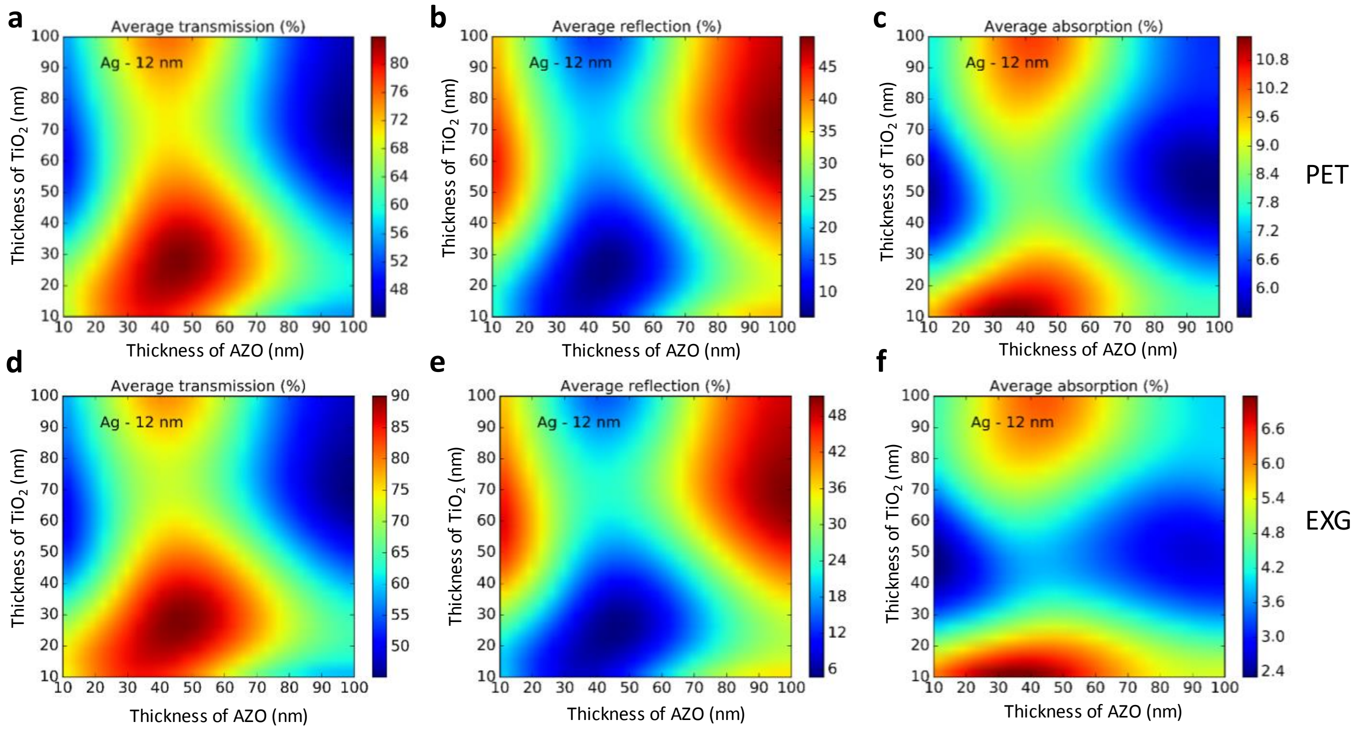

Supplementary Figure 2. Optical Simulation of AR-TC on PET (top raw) and EXG (bottom raw). Simulated (**a,d**) transmission, (**b,e**) reflection and (**c,f**) absorption of AR-TC for different TiO_2 and Al doped ZnO (AZO) thicknesses for Poly Ethylene Terephthalate (PET) polymer and Corning Eagle XG glass substrates. For all the structures, the Ag film thickness is kept constant at 12 nm. The transmission, reflection and absorption include the substrate contribution, i.e. they refer to the whole TC on substrate structure, and are average values over 400 to 700 nm wavelength range. The PET substrate was measured to be slightly absorbing. This was taken into account in the corresponding simulations through the extinction coefficient (imaginary part of refractive index in Supplementary table 1).

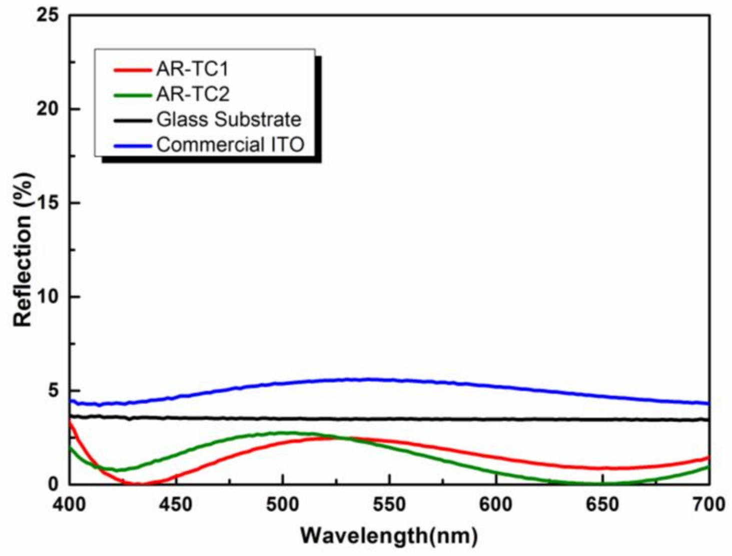

Supplementary Figure 3. Single side reflection measurements comparison. Experimental values of single side reflection of the proposed AR-TC compared with commercial ITO and fused silica glass substrate over the visible wavelength range (400-700 nm), obtained by index matching a completely absorbing material (black glass) to the back surface.

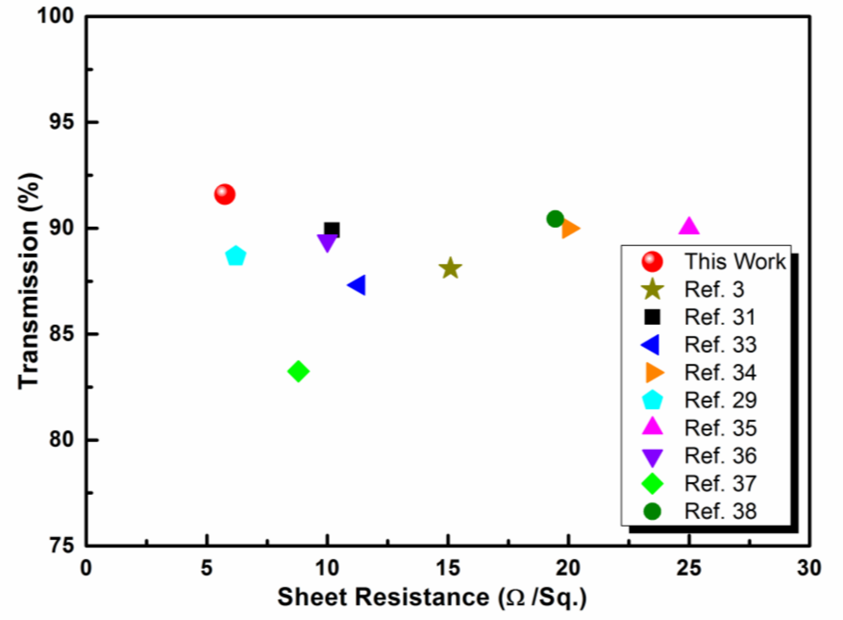

Supplementary Figure 4. AR-TC performance comparison with literature. Transmission at 550nm as a function of electrical sheet resistance of the proposed AR-TC and other TCs from the literature. All the reported values include substrate contribution (two side measurements).

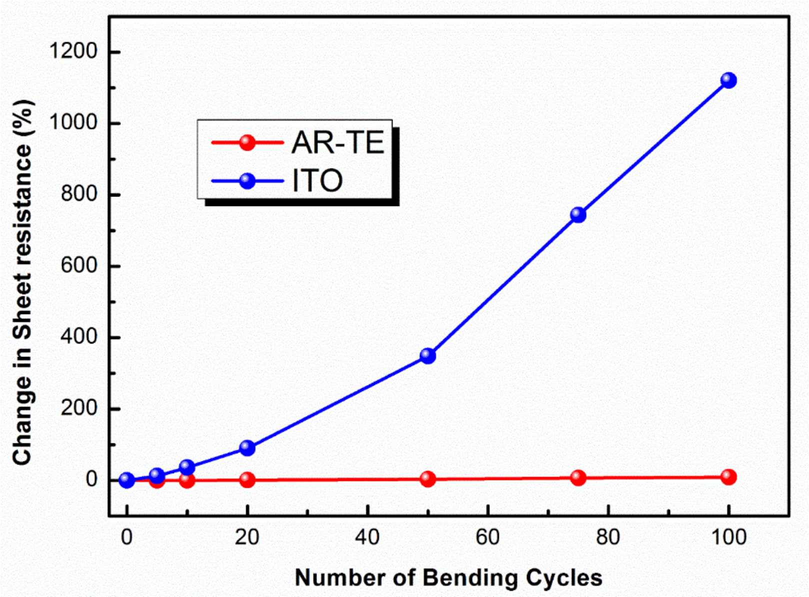

Supplementary Figure 5. Mechanical flexibility performance of AR-TC. Change in sheet resistance of AR-TC after bending as a function of bending cycle compared with conventional ITO. Maximum and minimum bending radii are 10 and 3 mm, respectively.

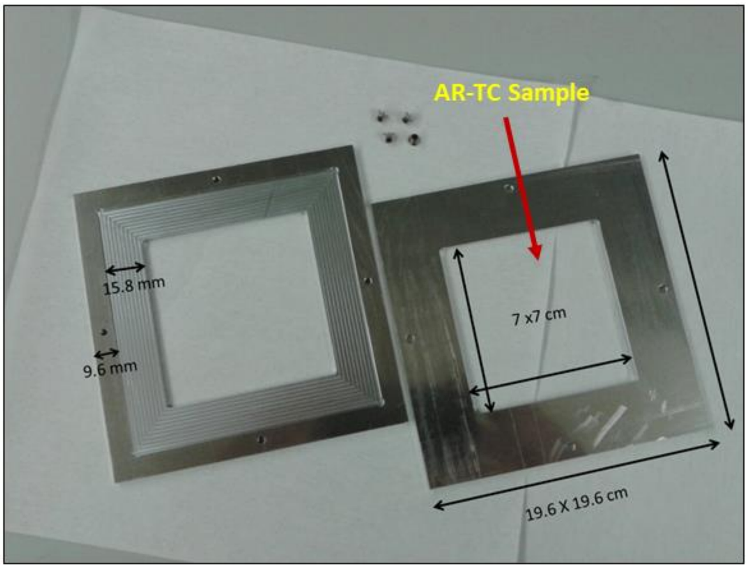

Supplementary Figure 6. EMI shielding measurement. AR-TC sample prepared and embedded in an Al frame for shielding efficiency measurement.

Substrate Material	Refractive Index	Substrate Transmission, T_S (%)	Total Transmission, T_{TOT} (%)	TC Transmission, T_{TC} (%)	Optical Loss, OL (%)
**Fused Silica**	**1.46**	**93.16**	**91.6**	**98.33**	**1.56**
**Corning Eagle XG**	**1.51**	**91.08**	**89.23**	**97.97**	**1.85**
**PET**	**1.65 + 1.49e-5 i**	**85.6**	**82.43**	**96.30**	**3.17**

Supplementary Table 1. Experimental comparison of AR-TC on different substrate materials. The reported values for all the transmission and optical loss are averaged over the visible range (400-700nm). Note that the total transmission in the table, T_{TOT}, includes both the TC (T_{TC}) and substrate (T_{S}). In fact we measured independently T_{TOT} (substrate with TC) and T_{S} (only substrate without TC) and calculated $T_{\mathrm{TC}} = T_{\mathrm{TOT}}/T_{\mathrm{S}}$. The optical loss, OL, is calculated as OL = T_{TOT} - T_{S}. Structures for PET and EXG were not fully optimized. PET was also highly reflective (high refractive index) and slightly absorbing.
